# Strengthening therapeutic adherence and pharmacovigilance to antimalarial treatment in Manaus, Brazil: a multicomponent strategy using mHealth

**DOI:** 10.1186/s12936-022-04047-3

**Published:** 2022-01-29

**Authors:** Diego Macías Saint-Gerons, Sheila Rodovalho, Ádila Liliane Barros Dias, André Lacerda Ulysses de Carvalho, Andrea Beratarrechea, Wuelton Marcelo Monteiro, Myrna Barata Machado, Cristiano Fernandes da Costa, Marcelo Yoshito Wada, Márcia Helena Maximiano Faria de Almeida, Rayanne Silva de Matos Fonseca, Jady Shayenne Mota Cordeiro, Alinne Paula Rodrigues Antolini, João Altecir Nepomuceno, Karen Fleck, Fernanda Simioni Gasparotto, Marcus Lacerda, Robin Rojas-Cortés, Shanthi Narayan Pal, Analía I. Porrás, María de la Paz Ade, José Luis Castro

**Affiliations:** 1 Department of Medicine, University of Valencia, INCLIVA Health Research Institute and CIBERSAM, Valencia, Spain; 2 Unit of Medicines and Health Technologies (MT), Dep. of Health Systems and Services (HSS), Pan American Health Organization (PAHO/WHO), Washington, USA; 3 Programa de Pós-Gradação Em Medicina Tropical, Universidade Do Estado Do Amazonas, Manaus, AM Brazil; 4 Communicable Diseases and Environmental Determinants of Health (CDE), Pan American Health Organization (PAHO/WHO), Brasília, Brazil; 5 Unit of Medicines and Health Technologies (MT), Dep. of Health Systems and Services (HSS), Pan American Health Organization (PAHO/WHO, Brasília, Brazil; 6 Institute of Clinical Effectiveness and Health Policy (IECS), Buenos Aires, Argentina; 7 Fundação de Medicina Tropical Dr. Heitor Vieira Dourado, Manaus, Brazil; 8 Universidade do Estado do Amazonas, Manaus, Brazil; 9 State of Amazonas Health Surveillance Foundation, Amazonas State Health Secretariat, Manaus, Amazonas Brazil; 10 General-Coordination for Surveillance of Zoonoses and Vector-Borne Diseases, Secretariat of Health Surveillance, Ministry of Health, Manaus, Brazil; 11 Environmental Surveillance Management - Department of Health, Manaus, Amazonas Brazil; 12 Pharmacovigilance Office (GFARM), Brazilian Health Regulatory Agency (Anvisa), Brasília, Brazil; 13 Instituto Leônidas & Maria Deane, Fiocruz, Manaus, Brazil; 14 World Health Organization Headquarters, Geneva, Switzerland; 15 Department of Communicable Diseases and Environmental Determinants of Health, Pan American Health Organization (PAHO/WHO), Washington, USA

**Keywords:** Primaquine, Adherence, Pharmacovigilance, mHealth, SMS

## Abstract

**Background:**

Public health initiatives for improving adherence to primaquine based regimens and enhancing effective pharmacovigilance are needed to support the efforts for malaria elimination in real world conditions.

**Methods:**

A multicomponent patient-oriented strategy using a Smart Safety Surveillance (3S) approach including: (1) educational materials for treatment counselling and identification of warning symptoms of haemolytic anaemia; (2) an mHealth component using Short Message Service (SMS) treatment reminders and (3) development and implementation of follow-up phone surveys three days after treatment completion, using a web-based platform linked to the local information system of malaria. Adherence was measured using the Morisky Medication Adherence Scale. Self-reported events were registered using a structured questionnaire and communicated to the Brazilian Health Regulatory Agency.

**Results:**

Educational materials were disseminated to 5594 patients, of whom 1512 voluntarily entered the mHealth component through the local information system; 7323 SMS were sent, and 1062 participants completed a follow-up survey after treatment. The mean age of patients was 37.36 years (SD 13.65), 61.24% were male, 98.54% were infected with. *Plasmodium vivax* and 95.90% received a short regimen of chloroquine plus primaquine (CQ + PQ 7 days), as per malaria case management guidelines in Brazil. From the 1062 surveyed participants 93.31% were considered adherent to the treatment. Most of the patients (95.20%) reported at least one adverse event. Headache, lack of appetite and nausea/vomiting were the most frequently reported adverse events by 77.31%, 70.90% and 56.78% of the patients respectively. A quarter of the patients reported anxiety or depression symptoms; 57 (5.37%) patients reported 5 to 6 warning symptoms of haemolytic anaemia including jaundice and dark urine in 44 (4.14%). Overall, three patients presenting symptoms of haemolytic anaemia attended a hospital and were diagnosed with G6PD deficiency, and one had haemolysis. All of them recovered.

**Conclusions:**

Under real world conditions, a multicomponent patient-oriented strategy using information and communication technologies allowed health care providers to reinforce treatment adherence and enhance safety surveillance of adverse events associated with regimens using primaquine. Active monitoring through phone surveys also reduced under-reporting of ADRs. This approach is low-cost, scalable and able to support prioritized activities of the national malaria programme.

**Supplementary Information:**

The online version contains supplementary material available at 10.1186/s12936-022-04047-3.

## Background

The burden of malaria has declined since the mid-2000s in terms of age-standardized incidence rates in the world [1]. However, in the Region of the Americas, it is estimated that malaria caused 889,000 cases in 2019 [2]. In Brazil, approximately 99.5% of malaria cases occur within the Amazon Region, of which 87.1% were caused by *Plasmodium vivax* in 2019 [3, 4] with a greater proportion of the cases diagnosed in males [5]. The treatment of choice for infections caused with *P. vivax* is a regimen treatment of chloroquine plus primaquine since a radical cure can be achieved through latent liver phases (hypnozoites) elimination [6]. Failure to clear the hepatic stages has also been reported with (use of) primaquine, and has been associated with lack of treatment adherence [7]. Moreover, like any other drug, antimalarials have the potential to cause adverse effects [8]. Acute haemolytic anaemia is a dose-dependent adverse reaction associated with primaquine which can be life-threatening in individuals with glucose-6-phosphate dehydrogenase (G6PD) deficiency, a genetically X-linked disorder [9]. However, spontaneous reporting rates of adverse drug reactions associated with antimalarials, including haemolytic anaemia, are very low [10].

Public health policies encourage strategies for improving adherence to antimalarial medications and enhancing effective pharmacovigilance for primaquine based regimens in where rapid diagnostic test for detection of G6PD deficiency are not in place [9]. We applied a Smart Safety Surveillance (3S) pharmacovigilance risk prioritization approach [11] to strengthen therapeutic adherence, patient education and pharmacovigilance of the anti-malarial treatment through three components: (1) educational packaging with information about both the treatment and the identification of warning symptoms of haemolytic anaemia; (2) an mHealth component to send treatment reminders via SMS and (3) safety surveys and event reporting using electronic communication with Regulatory Authorities.

## Methods

### Participating institutions and centers

A collaborative project using a Smart Safety Surveillance (3S) pharmacovigilance risk prioritization approach was developed with the participation of the National Malaria Control Programme (Ministry of Health of Brazil), the Brazilian Health Regulatory Agency (*Agência Nacional de Vigilância Sanitária*—ANVISA) and the Pan-American Health Organization (PAHO). The project was coordinated at the Departmental level with the Health Secretariat of Amazonas, The Tropical Medicine Foundation of Amazonas, Dr Heitor Vieira Dourado (tertiary care) and 74 health units in the Manaus region. Health units are specialized centers in the treatment of malaria and other tropical diseases and dispense treatments to their patients after confirmed diagnoses of uncomplicated malaria. All the centers register patients receiving treatment in the Brazilian National Malaria Surveillance System (SIVEP-Malária). The SIVEP Malária is an online surveillance system from the Ministry of Health that records all notified cases of malaria in the Amazon region of Brazil since 2003 [12]. Training visits to the health units were arranged in order to ensure that the materials were provided to the patients, and that the inclusion criteria for further participation in the adherence programme were explained. The project included patients initiating treatment from November 4^th^ 2019 to December 5^th^ 2020.

## Multicomponent strategy

### Educational material

**Fig. 1:**
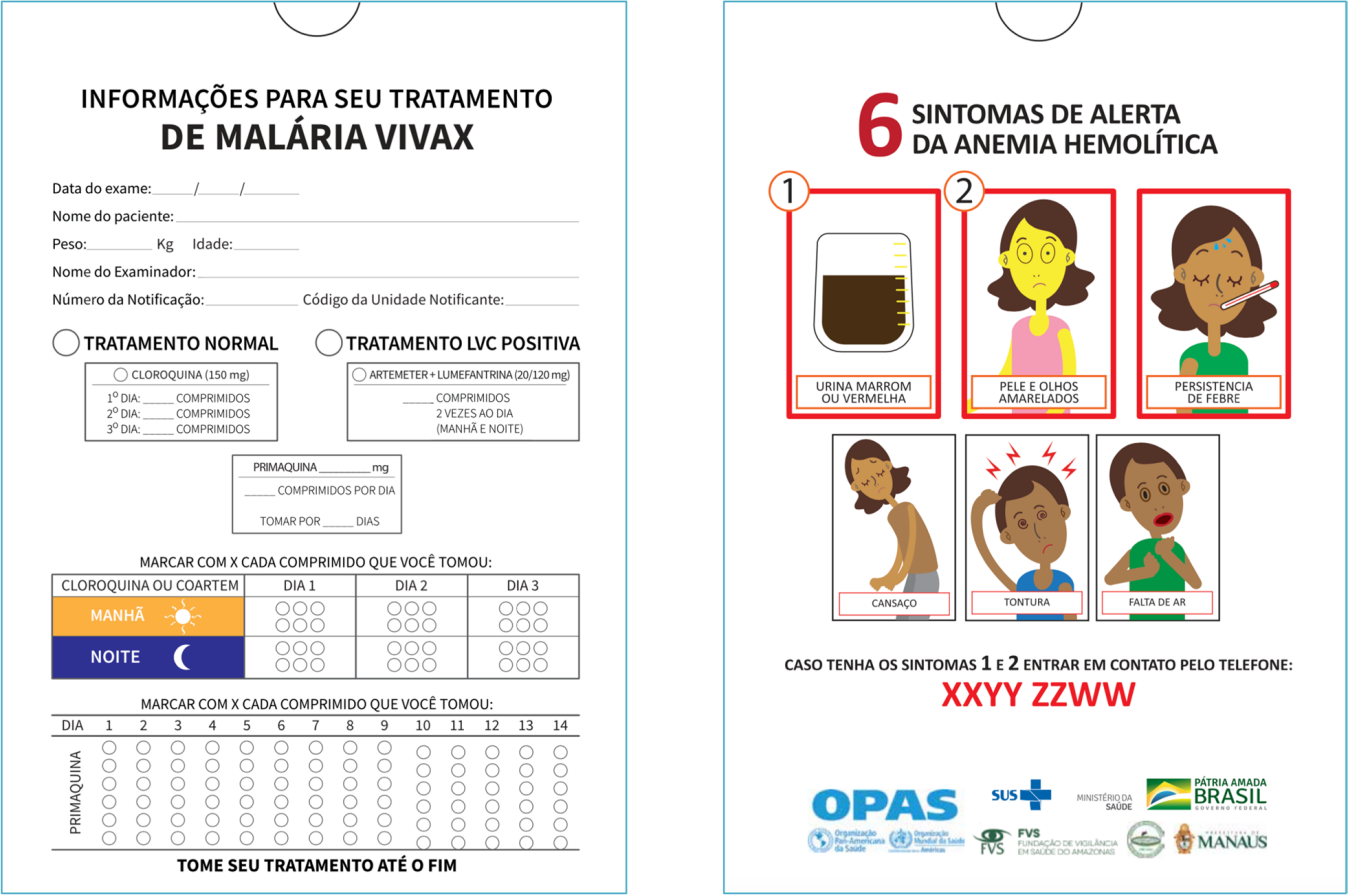
Educational packaging with instructions for the patients

An envelope designed to package/contain the tablets of anti-malarial treatment was provided to all the patients initiating treatment. The envelope was A3 size and printed on two sides. The front included instructions on the type and number of tablets to take and the dosage. The back included six illustrations of haemolytic anaemia warning symptoms and an emergency phone number (Fig. 1) [9].

### Web based platform

A web-based platform using REDCap (v8.11.10) electronic data capture tools (https://www.project-redcap.org) provided the necessary integration of the health intervention mobile component. Briefly, REDCap is a web-based software tool developed at the Centre for Clinical and Translational Research at Vanderbilt University in Nashville, Tennessee, USA. REDCap is a tool for the design and development of electronic data capture used by more than five thousand institutions in 144 countries. Details have been published elsewhere [13, 14].

The web-based platform facilitates the importation of the necessary participant data from SIVEP and the analysis of the information captured. Imported data included: patient name, telephone number, age, gender, ethnicity, malaria diagnosis, treatment regimen, information about the initiation of treatment, and informed consent. The following inclusion criteria were used to send text messages to the patients and for the follow-up phone calls: (1) Patient aged ≥ 16 starting treatment for uncomplicated malaria; (2) with a mobile phone for personal use and consenting to receive free SMS and follow-up calls. Only patients who met these criteria were imported into the web-based platform. To ensure the confidentiality of the information, an isolated project server was used, a secure layer protocol was implemented in order to secure the connection through Secure Sockets Layer (SSL), and personal access credentials (username and password) were required for project collaborators. Data from the treated patients were imported into the web platform daily from the local platform SIVEP through files in MS Excel. Personal information was de-identified and encrypted in the system for data analysis.

### mHealth component

The patient information collected in the health units was assembled using specific algorithms based on the treatment regimen available. In addition, a set of SMS text messages was developed according to the 4 potential treatment regimens available. The total number of messages ranged from 3 planned SMS for the shorter regimens (7 days) to 7 for the longer regimen (14 days) and they were structured as follows: (1) welcome message; (2) adherence reminder; (2) encouraging/positive adherence reminder (countdown); (3) safety reminder; (4) end message (Additional file 1: Tables S1, S2).

The web server running the REDCap software was linked to an Android cell phone running scripts in the automated application previously installed on the phone. A dedicated cell phone was permanently connected to the Internet, with access to the server and with an active line with available credit. A basic monthly plan of 49.99 R$ (9.19 USD) was contracted which allowed the sending of unlimited free SMS. The capacity of the cell phone was set to a maximum of 600 messages per day for adequate response time. Communication of the phone with the server was possible through an Application Programming Interface (API) connected to a specifically developed REDCap External Module.

In the server, the messages were generated automatically as the patients were entered, according to the participant data. The cell phone asked the server for the next message to send, sent it and then reported the status to the server. Receiving SMS was cost-free to the participants. The SMS operating system was adapted by the Institute of Clinical and Health Effectiveness (IECS) [15].

### Phone surveys and electronic communication of events

A structured questionnaire was developed in order to gather information about patient´s comorbidities, co-medication and self-reporting of adverse events during the treatment, including: warning symptoms of haemolytic anaemia, gastrointestinal complaints, skin complaints, psychiatric and neurological symptoms, and issues with heart rhythm, hearing and balance. A self-report adherence scale using the Morisky Medication Adherence Scale (MMAS-4) was also included. The MMAS-4 comprises four questions with a dichotomous answer (yes/no) which reflects the conduct of the patients regarding medication adherence. The patient is considered adherent if the 4 questions are answered correctly [16].

Responses to the questionnaire were registered in the web-based platform, which also provided the pollsters with a calendar for the phone calls. Phone calls were carried out by a trained team of pollsters 3 days after the end of each patients' treatment. Pollsters made a maximum of five contact attempts on the day corresponding to the survey for each patient. In the case of a patient being hospitalized or attending a health unit during the treatment, efforts were made to complete the clinical information in collaboration with the referral hospital or health unit.

A trained person manually registered and reported to the Brazilian Health Regulatory Agency Individual Case Safety Reports (ICSRs) using the web-based system VigiMed, following international standards for transmitting medicine adverse event reports and coding adverse events and any other medical terms included at the ICSRs. VigiMed it is in direct connection with VigiBase, the global database of ICSRs [17]. Supplementary to manual reporting of events, a complementary web-based module exported data from the events recorded in the web-based platform. When an event was recorded, an Individual Case Safety Report (ICSR) using the E2B format was generated for the patient. E2B is an international standard for transmitting medicine adverse event reports specified by the International Conference on Harmonization of Technical Requirements for Registration of Pharmaceuticals for Human Use (ICH) [18]. The adverse events were coded using the Medical Dictionary for Regulatory Activities (MedDRA) (Additional file 1: Table S3). The ICSRs were reported electronically each week in an XML file sent to a designated focal point in the Regulatory Agency (Additional file 1: Fig. S1).

### Statistical analysis

Information from the phone surveys and text messages registered on the platform was used to assess how the intervention was carried out. The ratio between SMS programmed and SMS effectively sent served as a measure of the mobile phone’s performance. Patient adherence was evaluated based on the proportion of patients classified as adherent according to the Morisky-Green scale MMAS-4 [16]. The main demographic characteristics and self-reported events were descriptively summarized using proportions, median (range) or mean (standard deviation) as appropriate. The Chi square test or t-test was used to test the consistency of the results and explore the potential differences between contacted and not contacted patient groups. P values < 0.05 were considered statistically significant. All analyses were conducted using Stata version 14 (StataCorp LP, College Station, Texas, USA).

## Results

**Fig. 2:**
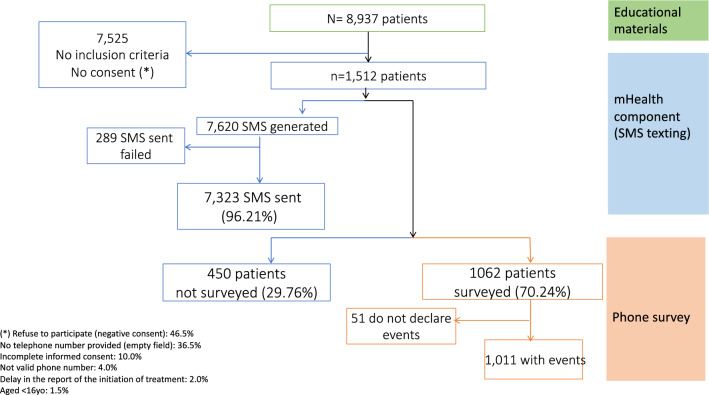
Participating patients flow chart

Educational materials were disseminated to 8937 patients, of whom 1512 met the criteria to receive the mHealth component (Fig. 2). The mean age of the patients was 37.36 (SD: 13.65) years; most of the patients were male (61.24%), pardo (mixed) ethnicity (80.49%) and infected with *P. vivax* (98.54%); 1062 completed a phone survey of safety and adherence. The causes identified for non-answering were: invalid phone number (21.33%), call answered by a person other than the patient (15.33%), automatic response of the telephone operator (absent or out of range) (4.89%), rejected call or no answering (7.56%); the reason for non-answering was unknown in 50.89% cases. There were no statistically significant differences in the basal characteristics (age, gender, ethnicity, malaria type, type of treatment) between contacted and not contacted groups (Table 1). From the surveyed patients, the most common treatment regimen was the “short standard” (seven days) with chloroquine for three days (10 mg / kg on day 1 and 7.5 mg / kg on days 2 and 3) + Primaquine 0.50 mg / kg weight for 7 days (30 mg per day) (95.52%); Of these, 1011 (95.20%) patients reported at least one event; 151 (14.22%) patients had one or more comorbidities, with hypertension and diabetes being the most prevalent ones; 440 (41.43%) patients received other concomitant drugs. Dipyrone and paracetamol were the most frequently used co-medications, by 222 (20.90%) and 75 (7.06%) patients respectively. Headache was the most frequently reported event in 821 (77.31%) patients, followed by lack of appetite (anorexia) in 753 (70.90%) patients and gastrointestinal events: 603 (56.78%) patients reported nausea or vomiting, 463 (43.60%) gastralgia and 339 (31.92%) diarrhea; depressive and anxious moods were reported by 245 (27.07%) and 243 (22.88%) patients, respectively; 66 (6.21%) patients reported visual hallucinations. Tachycardia was reported by 345 (32.49%) respondents.

**Table 1:** Baseline characteristics of the patients receiving educational materials and SMS texting (mHealth)

Characteristics	Non-contacted group n = 450	Contacted group n = 1062	Total N = 1512
Age in years^a^			
16–25	104 (25.11)	240 (22.60)	344 (22.75)
26–35	129 (28.67)	267 (25.14)	396 (26.19)
36–45	97 (21.56)	260 (24.48)	357 (23.61)
46–83	120 (26.67)	295 (27.78)	415 (27.45)
Gender (% male)^a^	285 (63.33)	641 (60.36)	926 (61.24)
Ethnicity (%)^a^			
White	7 (1.56)	37 (3.48)	44 (2.91)
Pardo (mixed)	365 (81.11)	852 (80.23)	1217 (80.49)
Indigenous	1 (0.22)	0 (0.00)	1 (0.07)
Black	11 (2.44)	40 (3.77)	51 (3.37)
Yellow	3 (0.67)	9 (0.85)	12 (0.79)
Unknown	63 (14.00)	124 (11.68)	187 (12.37)
Type of malaria^a^			
P. Vivax	444 (98.67)	1,046 (98.49)	1490 (98.54)
P. Falciparum	5 (1.11)	8 (0.75)	13 (0.96)
Mixed infection/other	1 (0.22)	8 (0.75)	9 (0.60)
Treatment regimen^a^			
Long standard regimen CQ + PQ^b^	19 (4.22)	23 (2.17)	42 (2.78)
Short standard regimen QC + PQ^**c**^	425 (94.44)	1025 (96.52)	1450 (95.90)
Combined therapy AT + PQ^d^	5 (1.11)	7 (0.66)	12 (0.79)
Mixed malaria scheme^f^	1 (0.22)	7 (0.66)	8 (0.53)

CQ: chloroquine; PQ: primaquine; AT: artemisin; ^a^ No statistically significant difference between contacted and uncontacted groups (P ≥ 0.05); ^b^ Long standard regimen: Chloroquine 25 mg / kg weight divided in 3 days (1500 mg adult dose) + Primaquine 0.25 mg / kg weight for 14 days (15 mg per day); ^c^ Short standard regimen: chloroquine for three days (10 mg / kg on day 1 and 7.5 mg / kg on days 2 and 3) + Primaquine 0.50 mg / kg weight for 7 days (30 mg per day); ^**d**^ Combined therapy based on Artemisin + Primaquine (single dose) 3 days; ^f^ Mixed malaria scheme: Artemisinin + Primaquine 2 tablet of 15 mg (single daily dose) for 7 days (double dose)

Overall 57 (5.37%) patients declared that they had experienced 5–6 warning symptoms of haemolytic anaemia. Jaundice and dark urine—the most specific warning symptoms—were reported by 419 (39.45%) and 71 (6.69%), respectively; 44 (4.14%) patients reported both symptoms. Reported event rates are available in Table 2. Three patients with symptoms of haemolytic anaemia contacted the phone number provided in the educational materials, and were directed to a referral hospital. The patients had G6PD deficiency and one of the them presented with haemolysis. None of them required erythrocyte transfusion and all recovered (Table 3). In total 57 (5.37%) patients reported having attended a hospital or health unit during the course of treatment. The most frequent reasons to attend health care centers (not necessarily related to the treatment) were increased malaria symptoms (fever, headache), cutaneous and gastrointestinal disturbances (Additional file 1: Table S4). Of the surveyed patients, 991 (93.31%) were considered adherent. After completing the treatment, 1032 (97.45%) of the patients believed their malaria went away and experienced health improvement.

**Table 2:** Comorbidities, comedication, self-reported events and adherence in patients contacted by phone using a follow-up survey

Patient condition	n (%)
Comorbidities	151 (14.22)
Hypertension	83 (7.82)
Diabetes	58 (5.46)
Asthma	8 (0.75)
HIV	7 (0.66)
Other disease	22 (2.07)
Concurrent treatments	
Dipyrone	222 (20.90)
Paracetamol	75 (7.06)
Renin–angiotensin–aldosterone system inhibitors	36 (3.39)
Orphenadrine	28 (2.54)
Oral antidiabetics	19 (1.79)
Ibuprofen	18 (1.69)
H1-antihistamines	8 (0.75)
Diuretics	8 (0.75)
Dimenhydrinate	6 (0.56)
Number of symptoms suggestive of hemolytic anaemia	
No symptoms	217 (20.43)
1–2 Symptoms	481 (45.29)
3–4 Symptoms	307 (28.91)
5–6 Symptoms	57 (5.37)
Type of symptom suggestive of hemolytic anaemia	
Dark urine	71 (6.69)
Yellow skin and / or eyes	419 (39.45)
Fever	190 (17.89)
Back pain	511 (48.12)
Dizziness	586 (55.18)
Shortness of breath	276 (25.99)
Gastrointestinal changes	
Stomach ache	463 (43.60)
Nausea or vomiting	603 (56.78)
Diarrhea	339 (31.92)
Skin disorders	
Itchy or burning skin	287 (27.02)
Red patches or patches on the skin	74 (6.97)
Psychiatric disorders	
Anxious mood	243 (22.88)
Depression mood	245 (27.07)
Hallucination, visual	66 (6.21)
Lack of appetite	753 (70.90)
Neurological disorders	
Headache	821 (77.31)
Agitation and tremors without control	111 (10.08)
Changes in heart rate	
Accelerated heartbeat	345 (32.49)
Ototoxicity	
Feeling of loss of balance	392 (36.91)
Ringing in the ear	130 (12.24)
Adherent to treatment	991 (93.31)

CQ: Chloroquine, PQ: primaquine

**Table 3:** Summary of the clinical characteristics of the patients referred to the hospital with suspected Acute Hemolytic Anaemia

	Age/Sex	Treatment regimen	Indication	Relevant comorbidities; comedication	G6PD deficiency	Haemolysis	Hemoglobin (g/dL)	Serum bilirubin, total/conjugated, mg/dL	Transaminases (U/L)	Hospitalization period until discharge (days)
Case 1	11/M	Short standard regimen QC + PQ	Mixed malaria (p.falciparum & vivax)	None; Dipyrone	Yes	No	12.06	1.57/ 0.50	AST: 56 ALT: 30	4 days
Case 2	35/M	Short standard regimen QC + PQ	Malaria p.Vivax	None; Dipyrone	Yes	Yes	12.83	4.65/1.72	AST: 231 ALT: 305	0 days
Case 3	24/F	Short standard regimen QC + PQ	Malaria p.Vivax	None; No	Yes	No	12.38	0.52/0.20	AST: 32 ALT:52	1 day

G6PD: glucose-6-phosphate dehydrogenase; AST, aspartate aminotransferase; ALT, alanine aminotransferase

## Discussion

This multicomponent strategy based on the utilization of educational materials, mHealth (text messaging intervention) and local information systems, enabled the use of complementary systems to reinforce adherence to, and enhance safety surveillance of, the anti-malarial treatment regimen and specifically the one with primaquine use for radical cure A high rate of adherence was found after educational materials and SMS were disseminated. This approach is low-cost and scalable.

In Latin America and Caribbean (LAC), cases of uncomplicated malaria caused by *P. vivax* are commonly treated on an outpatient basis, using unsupervised regimens including primaquine. Unsupervised treatment should be accompanied by health messages in order to improve adherence [19]. Low adherence to primaquine anti-malarial regimens has been associated with malaria relapses, treatment failure, and could be a contributing factor in potential drug-resistance [7, 20]. Previous studies have reported adherence rates of 58.8% and 62.2% in Ecuador (*P. vivax)* and Peru, respectively [21, 22]. In Brazil the number of patients included in four adherence studies ranged from 16 to 414 patients. Two self-reported adherence studies found adherence of 83.8% and 87.5% [23, 24]. A third study using a combination of self-reporting of adherence and counting of missing tablets the adherence found was 78.2%according to pill-counts and 86.4% according to self-report [25]. Other study with patients being treated for P. vivax in Brazil reported 86.4% of adherence in self-report adherence (interview) and counting of missing tablets [26]. In this project, the self-reported adherence was 93.31% (991 patients) for patients who received the packaging with educational materials plus the SMS texting.

Specialized packaging and messaging, including pictorial instructions, have been used in previous studies to increase the adherence to anti-malarial medications [27, 28]. Mobile health has also been used to reinforce patient adherence to treatment. Using text messaging intervention has the advantage over mobile apps of not requiring smartphones, being free for patients, not consuming patient mobile data and not requiring mobile internet or Wi-Fi internet connections. Previous randomized trials performed in Africa have shown increased adherence to anti-malarials associated with the use of SMS treatment reminders in adults [29, 30] but not among children [31]. However, no similar trials have been performed for anti-malarials in LAC. One pivotal factor limiting the effectiveness of SMS texting is the mobile phone infrastructure in the area [32]. There is country-to-country variability, but penetration in some countries like Brazil exceeds 100% [33]. In Manaus, four different mobile phone companies provide service, and there is basic coverage along the main rivers and road routes where small remote rural populations are concentrated. In these areas, the use of personal mobile phones has also increased notably during recent years [34].

Overall, self-reported events were mild and patients were able to complete the treatment. Nausea, vomiting, anorexia and headache were very common. The rate of self-reported events was similar to a previous study [35]. However, a quarter of the patients reported experiencing depression or anxiety during the treatment and 6% reported visual hallucinations. Anti-malarials can affect the central nervous system, causing adverse mental and neurological effects [36]. Visual hallucinations and delusions have been reported with chloroquine but are considered rare [37]. Moreover, extrapyramidal syndromes and reversible tremors have also been also documented with chloroquine [38]. A meta-analysis of studies of patients exposed to anti-malarials found a lower prevalence of mental and neurological events associated with primaquine (0.7%) than with chloroquine (7.1%) [39]. These findings should be interpreted with caution due to the very high heterogeneity found (I^2^ = 97.8%). Recent studies have suggested a two-way association between malaria and mental disorders [40, 41]. In the absence of large comparative studies with adequate control for confounding factors, it is difficult to differentiate the neuropsychiatric effect of primaquine, chloroquine or its combined use, from that of malaria itself. Nevertheless, strategies that consider baseline evaluations and strengthen the management of mental health during the acute treatment of malaria should be discussed in the planning of strategic actions by the Malaria Programme. The high rate of suggestive tachycardia reported by patients also merits further investigation [42].

For more than six decades it has been known that patients with G6PD deficiency or a family history of favism (hereditary G6PD deficiency) have an increased risk of primaquine-associated haemolytic anaemia [43, 44]. The incidence of primaquine associated haemolytic anaemia is thought to be rare; however, to date, risk quantification remains imprecise. Figures have been extrapolated from ecological studies of the mass use of primaquine in US soldiers in the Korean War, the programmes of the Union of Soviet Socialist Republics (USSR) during the 1950s, and the radical mass preventive treatment regimens used in some 28 million people in the Chinese Province of Jiangsu in the 1970s [8]. In the latter, population incidence of haemolysis has been estimated to be 9.3/100,000 [45]. The global prevalence of G6PD deficiency has been estimated at 8% of the population [46]. In the Western Amazonas, the prevalence of G6PD deficiency was estimated to be 4.5% [47]. In this survey 5.4% patients reported experiencing 5 to 6 warning symptoms of haemolytic anaemia and 4.1% reported jaundice and dark urine. Mild haemolytic anaemia symptoms can resolve with time and without treatment. However, close medical attention is advised for those patients, since haemolytic anaemia is a serious adverse reaction that can lead to death. During the study period, three patients were referred to the hospital with symptoms of Acute Haemolytic Anaemia, one of whom presented with haemolysis; this accounted for a cumulative incidence of 3.4 cases per 10,000 treated patients. A systematic review of published studies (any design) identified 47 cases of primaquine-induced haemolysis in LAC, of which 23 cases originated in Brazil [48]. Under diagnosis was recognized by the authors as a possible explanation for the low number of cases identified. A previous case series study performed in Manaus described 17 hospitalized patients treated with chloroquine and primaquine who presented jaundice, severe anaemia and other complications but causality was judged to be unclear [49].

However even in the case of suspicion it is important reporting of all suspected adverse drug reactions (ADR) on all drugs through the national reporting systems. Under-reporting of suspected ADR to the regulatory authorities delays the detection and identification of safety problems, making it difficult to interpret risk or take actions to preserve public health [50]. Several hypotheses for the under-reporting of ADR associated with anti-malarials have been proposed. In particular, the potential occurrence of ADR in remote areas outside the direct scrutiny of health professionals—who mostly contribute to the reporting of ADRs—could be a strong barrier for ADR reporting [51]. In this context, communicating patient-reported adverse reactions can be an important complementary source of information, adding new information and perspective about ADRs especially in daily life, in a way otherwise unavailable [50, 52].

As in any other study using phone surveys, self-reporting bias and recall bias cannot be ruled out. However, the Morisky-Green Scale used in the study has been validated as an indirect self-reporting method to measure medication adherence [16]. On the other hand, a short recall period was used in the study since the longest treatment duration was two weeks, and the most frequent regimen lasted one week. Moreover, the onset of haemolytic anaemia is considered to be acute, occurring generally within the first week of treatment. Monitoring warning symptoms of haemolytic anaemia is a recommended precautionary measure for the early detection of haemolytic anaemia according to the prescription information in the primaquine label and the programmatic malaria programme guidelines [9]. However, the appearance of symptoms does not necessarily mean an occurrence of a clinical case of haemolytic anaemia. Since some of the warning symptoms can be unspecific (i.e. fever, back pain, dizziness) the presence of simultaneous warning symptoms (5–6 symptoms) or specific concurrent symptoms such as dark urine or jaundice, as a proxy for the risk of haemolytic anaemia was analysed. Recruitment was limited due to the COVID-19 emergency, which caused reductions in the capacities and work force of health care facilities as well as imposed “stay at home” measures for the community. Delays in patient data entry into local systems and failures in data quality (i.e.: typing errors, missing data) should be recognized as the main limiting factors for recruitment to the mHealth. Strategies for encouraging patients to participate should be considered in the future, perhaps emphasizing the lack of cost of the SMS component for patient confidence and reinforcing the capture of the telephone number in the local sites of care.

Increasing the capacity to implement digital health, and in particular mHealth, has been prioritized in the 2030 Agenda for Sustainable Development, as it could play a major role in accelerating progress towards achieving universal health coverage, including ensuring access to quality health services [53]. In this study under real world conditions, routine data collection from local information systems was enhanced using complementary systems to improve safety surveillance and reporting of adverse events associated with treatment regimens using primaquine. However, combined strategies based on synergistic components are recommended to overcome the potential technical limitations of using digital health exclusively.

## Conclusion

Innovative approaches will be needed for the safety surveillance of current and upcoming drugs, which present safety concerns including dose-dependent haemolysis in G6PD-deficient individuals [54, 55]. This approach is low cost, scalable and able to support prioritized activities of the national programme of malaria.

## Supplementary Information

**Additional file 1.** Including information about the schedule and the exact wording of the SMS messages by type of treatment, MedDRA coding of pre-specified events, reasons declared by the patients for attendance to a hospital or health unit during treatment, checklist of symptoms of acute haemolytic anaemia and diagram of the multicomponent strategy implementation.

## Data Availability

The datasets during and/or analysed during the current study available from the corresponding author on reasonable request.
