# Supplementary material for: Strengthening therapeutic adherence and pharmacovigilance to antimalarial treatment in Manaus, Brazil: a multicomponent strategy using mHealth
Source: Malar J. 2022 Jan 29;21:28. doi: 10.1186/s12936-022-04047-3 (PMC8800548; doi:10.1186/s12936-022-04047-3)
Supplement: Supplementary file 1 — Additional file 1. Including information about the schedule and the exact wording of the SMS messages by type of treatment, MedDRA coding of pre-specified events, reasons declared by the patients for attendance to a hospital or health unit during treatment, checklist of symptoms of acute haemolytic anaemia and diagram of the multicomponent strategy implementation. [file 12936_2022_4047_MOESM1_ESM.docx]

Additional file 1: **Appendix**

**Table s1. Type of SMS messages**

| Type of message | Code | Text [Portuguese] |
| --- | --- | --- |
| Welcome | B | Olá [nome], seja bem vindo (a) ao sistema de mensagem de texto gratuito para te lembrar de tomar seu tratamento de malária” |
| Adherence reminder | T | T1 :“Recuerde continuar con su tratamiento según instrucción del personal de salud y aunque se sienta mejor no lo interrumpa”.  T2: Olá [nome] “Lembre-se de tomar seu tratamento esta noite !” (O aviso desta mensagem deverá ser programada para o envio no horário da tarde) |
| Encouraging adherence reminder (countdown) | R | R1: “Ânimo [nome], Faltam só 3 dias para terminar seu tratamento e você ficar curado da malária!”  R2: “Ânimo [nome], Faltam só 4 dias para terminar seu tratamento e você ficar curado da malária!” |
| Safey reminder | S | Olá [nombre], se você teve algum tipo de sintoma parecido com a informação que está no envelope do tratamento, entre em contato com o profissional de saúde no telefone do envelope! |
| Final | F | “Parabéns [nombre]! Hoje você completa seu tratamento! ” |

**Table s2. Planned frequency of SMS messages by type of treatment**

| Treatment regimen | Days of treatment | | | | | | | | | | | | | |
| --- | --- | --- | --- | --- | --- | --- | --- | --- | --- | --- | --- | --- | --- | --- |
|  | **0** | **1** | **2** | **3** | **4** | **5** | **6** | **7** | **8** | **9** | **10** | **11** | **12** | **13** |
| Chloroquine 25 mg / kg body weight divided over 3 days (1500 mg adult dose) + Primaquine 0.25 mg / kg body weight for 14 days (15 mg/day) | B |  | T |  | S |  | T |  |  | R2 |  | R |  | F |
| Chloroquine 25 mg / kg divided over 3 days (1500 mg adult dose) + Primaquine 0.50 mg /kg for 7 days (20 mg/day) + | B |  | T | R | S |  | F |  |  |  |  |  |  |  |
| Combined therapy of Artemisin + Primaquine (single dose) for 3 days | B | T2 | F |  |  |  |  |  |  |  |  |  |  |  |
| Combined therapy of Artemisin + Primaquine 15mgx2 tablets (single dose/day) for 7 days (double dose) | B |  | T | R | S |  | F |  |  |  |  |  |  |  |

B: Welcome message; T: adherence reminder; R: encouraging adherence reminder (regressive count) S: Safety reminder F: final reminder

**Table s3. MedDRA coding of pre-specified events**

| **Event name (portuguese)** | **Event name (english)** | **Level** | **MedDRA code** |
| --- | --- | --- | --- |
| Urina escura (cor de coca-cola) | Brown urine | LLT | 10058129 |
| Pele ou olhos amarelados | Jaundice | PT | 10023126 |
| Febre | Fever | LLT | 10016558 |
| Dor nas costas | Back Pain | PT | 10003988 |
| Tontura | Dizziness | PT | 10013573 |
| Falta de Ar | Dyspnoea NOS | LLT | 10013972 |
| Dor no estômago | Stomach ache | LLT | 10042076 |
| Enjoo ou vômito | Nausea and vomiting symptoms | HLT | 10028817 |
| Diarreia | Nausea | PT | 10028813 |
| Coceira ou ardor na pele | Pruritus | PT | 10037087 |
| Manchas ou placas vermelhas na pele | Rash | LLT | 10037844 |
| Ficou ansioso | Anxious mood | LLT | 10002875 |
| Ficou deprimido | Mood depression | LLT | 10027942 |
| Viu coisas | Hallucination, visual | PT | 10019075 |
| Falta de apetite | Decreased appetite | PT | 10061428 |
| Dor de cabeça | Headache | PT | 10019211 |
| Sentiu agitação e tremores sem controle (Convulsões) | Seizures | PT | 10039910 |
| Coração Acelerado | Tachycardia | PT | 10043071 |
| Sensação de perda de equilíbrio | Vertigo | PT | 10047340 |
| Zumbido no ouvido | Tinnitus | PT | 10043882 |
| Outro sintoma | Other | N/A | Free text |

LLT: Low-level term; PT: preferred term; N/A: Not available

**Table S4. Reasons declared by the patients for attendance to a hospital or health unit during treatment**

|  | n (%) |
| --- | --- |
| Increased malaria symptoms (fever, headache) | 22 (38.60) |
| Gastrointestinal disturbances | 5 (8.77) |
| Cutaneous disturbances | 5 (8.77) |
| Allergy | 4 (7.02) |
| Abdominal pain | 4 (7.02) |
| Thrombocytopenia | 2 (3.51) |
| Spleen surgery | 1 (1.75) |
| Unspecified surgery | 1 (1.75) |
| Blood in stool | 1 (1.75) |
| Dark urine | 1 (1.75) |
| Dysnea | 1 (1.75) |
| Hypertension | 1 (1.75) |
| Stains in the eyes | 1 (1.75) |
| Herpes | 1 (1.75) |
| Pregnancy detected | 1 (1.75) |
| Throat inflammation | 1 (1.75) |
| Work accident | 1 (1.75) |
| Cardiology consultation | 1 (1.75) |
| Hepatology consultation | 1 (1.75) |
| Request of treatment information | 1 (1.75) |
| Unspecified emergency | 1 (1.75) |

Table s5. **Checklist of symptoms of acute haemolytic anaemia**

| **Symptoms** |
| --- |
| Back pain  Dark (red or black) urine  Jaundice  Fever  Dizziness  Breathlessness |

Testing for G6PD deficiency for safe use of primaquine in radical cure of P. vivax and P. ovale. Policy brief. Geneva World Health Organization, 2016. Available in:

http://apps.who.int/iris/bitstream/10665/250297/1/WHO-HTM-GMP-2016.9-eng.pdf

**Figure S1. Diagram of the multicomponent strategy implementation**
